# Healthcare workers exposure risk assessment in the context of the COVID-19: a survey among frontline workers in Qazvin, Iran

**DOI:** 10.1186/s12913-023-09160-w

**Published:** 2023-02-15

**Authors:** Saeideh Moosavi, Peyman Namdar, Sakineh Moghaddam Zeabadi, Yousof Akbari Shahrestanaki, Mehran Ghalenoei, Mohammad Amerzadeh, Rohollah Kalhor

**Affiliations:** 1 Student Research Committee, School of Public Health, Qazvin University of Medical Sciences, Qazvin, Iran; 2 School of Medicine, Social Determinants of Health Research Center, Research Institute for Prevention of Non-Communicable Diseases, Qazvin University of Medical Science, Qazvin, Iran; 3 Department of Medical Emergencies, School of Paramedical, Social Determinants of Health Research Center, Research Institute for Prevention of Non-Communicable Diseases, Qazvin University of Medical Sciences, Qazvin, Iran; 4 Department of Pre-Hospital Emergency Medical Care, School of Paramedical Sciences, Qazvin University of Medical Sciences, Qazvin, Iran; 5 Department of Occupational Health Engineering, School of Public Health, Qazvin University of Medical Sciences, Qazvin, Iran; 6 Social Determinants of Health Research Center, Research Institute for Prevention of Non-Communicable Diseases, Qazvin University of Medical Sciences, Qazvin, Iran

**Keywords:** COVID-19, Healthcare workers, Exposure rate, Prevention and infection control

## Abstract

**Background:**

Healthcare workers perform various clinical procedures for COVID-19 patients facing an elevated risk of exposure to SARS-COV-2.This study aimed to assess the healthcare workers’ exposure to COVID-19 in Qazvin, Iran in 2020.

**Methods:**

We conducted this descriptive-analytical study among all healthcare workers on the frontline of exposure to COVID-19 in Qazvin province. We entered the participants into the study using a multi-stage stratified random sampling method. We utilized a questionnaire, "Health workers exposure risk assessment and management in the context of COVID-19 disease", designed by the World Health Organization (WHO) to collect data. We analyzed data using descriptive and analytical methods with SPSS software version 24.

**Results:**

The results showed that all participants in the study had occupational exposure to the COVID-19 virus. So of 243 healthcare workers, 186 (76.5%) were at low risk and 57 (23.5%) at high risk of COVID-19 virus infection. Also, from the six domains mentioned in the questionnaire, health workers exposure risk assessment and management in the context of COVID-19 disease, the mean score of the domain of the type of healthcare worker interaction with a confirmed COVID-19 patient, the domain of health worker activities performed on a confirmed COVID-19 patient, the domain of the adherence to infection prevention and control (IPC) during health care interactions, and the domain of the adherence to IPC when performing aerosol-generating procedures in the high-risk group were more than the low-risk group.

**Conclusion:**

Despite strict WHO guidelines, many healthcare workers are exposed at contracting COVID-19. Therefore, healthcare managers, planners, and policymakers can revise the policies, provide appropriate and timely personal protective equipment, and plan for ongoing training for staff on the principles of infection prevention and control.

## Background

COVID-19 is a viral respiratory disease detected in late December 2019 in Wuhan, China, in a group of patients with a severe respiratory infection [1–3]. A novel coronavirus causes COVID-19, scientifically named severe acute respiratory syndrome coronavirus 2 (SARS-COV-2) [4, 5]. Evidence suggests that the main transmission route is the inhalation of the patient's respiratory droplets. Still, contact with contaminated surfaces and transmission of the virus via the hands to the nose, mouth, and eyes can also lead to disease [6, 7].

Since the disease's incubation period is long, it is difficult to diagnose and interrupt the transmission of the disease from human to human [8]. The virus spreads rapidly among the population and leads to severe illness and even death in the elderly and people with a history of underlying disease [9, 10]. This has led to a public health emergency at the international level [11]. COVID-19, on the other hand, has disrupted the public health, economies, and social and personal lives of individuals around the world [9, 10]. Prevention (using personal protective equipment, hand washing, observing physical distancing, etc.) is still the best weapon against COVID-19 [10, 12].

Healthcare workers are at the frontline of the fight against COVID-19, so these people will be most exposed to the SARS-COV-2 virus [10, 13]. In case of high incidence among healthcare workers, the healthcare system will face a severe workforce shortage to deal with the epidemic. Therefore, all healthcare workers must observe the standard infection control precautions in dealing with each patient and providing service [14].

On February 19, Iran detected two cases of COVID-19 resulting in death, the first death of COVID-19 in West Asia, the Middle East, and North Africa. The virus spread rapidly in all provinces by March 5, 2020. Meanwhile, National COVID-19 Committee was established, led by the minister of health & medical education (MoHME). Subsequently, Provincial COVID-19 Committees were established led by the governor of the province and MoHME developed the first national guidelines for the prevention & control of the Coronavirus epidemic [15]. To date, large numbers of healthcare workers have been infected with COVID-19, according to reports from developed and underdeveloped countries [9, 10, 16]. For example, data from Italy indicates that 9% of their healthcare workers are infected with COVID-19 disease [17]. Regarding the high number of personnel involved in COVID-19 worldwide despite the relative use of personal protective equipment, assessing the clinical risk and exposure to the pathogen in healthcare workers can guide healthcare organizations’ managers and officials in better management and planning to perform safe risk management. Therefore, the World Health Organization (WHO) has developed a tool to measure the risk of exposure specifically for healthcare workers to COVID-19 disease. It can identify people with high and low exposure who are directly exposed to COVID-19 patients despite using personal protective equipment, hand washing, and observing physical distancing [18]. If exposure risk assessment is performed in healthcare centers and risk management responds immediately, high healthcare worker involvement can be prevented [9]. Since there is no information available on assessing the exposure of health workers in Iran to COVID-19, the present study aimed to assess the healthcare workers’ exposure to COVID-19 in Qazvin province in 2020.

## Methods

### Study design

This descriptive-analytical study evaluated the healthcare workers’ exposure to the COVID-19 disease in Qazvin province in 2020 (the beginning of the epidemic).

### Participants and sampling

We conducted this study among all healthcare workers in health centers, pre-hospital centers, and hospitals in Qazvin province in Iran in 2020. We entered the participants into the study using a multi-stage stratified random sampling method. We considered each hospital and pre-hospital emergency medical base as a category, and according to the required number of samples from each category, we randomly selected the samples. Based on the information obtained from the study of Ashinyo et al. (2020), we calculated the required number of samples using the formula below, which was 266 people ($\alpha =0.05$, $\beta =80\%$, $p=0/5,$  $\mathrm{d}=\left.0/06\right) \left(10\right)$.

$$n=\frac{{z}_\frac{a}{2}^{2}pq}{{d}^{2}}$$

During the research, 23 participants were excluded from the study due to unwillingness to cooperate. Finally, 128 nurses, 17 laboratory personnel, 44 emergency medicine service (EMS) personnel, nine radiologists, five anesthesiologist, five environmental health expert, seven medical doctors, 16 head nurses, 11 supervisors, and one faculty member participated in the study.

### Measurements

We used the questionnaire "Health workers exposure risk assessment and management in the context of COVID-19" designed by the WHO to categorize people according to the risk of exposure to the virus in different job situations [10, 19].

The questionnaire has two parts, the first part is related to the demographic information of healthcare workers, and the second part is the risk assessment survey adapted from WHO. The questions of the risk assessment section included COVID-19 risk assessment (2 questions), health worker activities performed on COVID-19 patients (4 questions), adherence to infection prevention and control (IPC) during health care interactions (7 questions), adherence to IPC when performing aerosol-generating procedures (6 questions) and accidents with biological material (1 question).

Healthcare workers answered "yes" to questions related to "COVID-19 risk assessment," and inquiries related to" activities performed on COVID-19 patients in healthcare centers" were considered as exposure to COVID-19. Also, in the risk classification, participants who did not respond "always, as recommended" in the questions "adherence to IPC during health care interactions" and "adherence to IPC when performing aerosol-generating procedures" were at high risk for COVID-19 infection. Finally, participants who did not respond "yes" to the question "did you have an episode of an accident with biological fluid/respiratory secretions?" were classified as high risk. All other responses were classified as "low risk for COVID-19". According to the people's responses to the questions, high risk people were identified, and exposure risk management programs can be applied [9, 18]. The internal consistency of the questionnaire was 0.89, which means that the tool has high reliability [9].

### Data analysis

In order to collect data, after determining the number of samples of each unit (Corona center hospitals and pre-hospital centers), questionnaires were randomly distributed among participants. We also explained to the participants that participating in the study is not mandatory. It should be noted that due to the conditions at that time, it was not possible to distribute the questionnaires physically, so the questionnaires were distributed electronically. After completing the questionnaires and collecting them, we analyzed data using descriptive and analytical statistics such as independent t-test and chi-square in SPSS software, version 24. A statistically significant level was considered *P* <0.05.

## Results

Table 1 shows the frequency distribution of demographic variables in the study participants (243 people). The majority of participants were females (65.8%, *N* = 160), had a bachelor (73.7%, *N* = 179), a nurse (52.7%, *N* = 128) working in the emergency medical base (18.1%, *N* = 44) and in Qazvin city (84.8%, *N* = 206). The results also showed that the mean age of participants was 34.95± 7.59, and the average work experience of healthcare workers was 11.16± 7.16.

**Table 1:** Frequency distribution of demographic variables among health care workers

Variable	Levels	Frequency	Percent
**Gender**	Male	83	34.2
	Female	160	65.8
**Highest level of qualification**	Diploma	4	1.6
	Associate degree	28	11.5
	Bachelor	179	73.7
	Master	25	10.3
	Doctor of medicine	7	2.9
**Type of health professional**	Nurse	128	52.7
	Laboratory personnel	17	7.0
	EMS	44	18.1
	Radiology	9	3.7
	Anesthesia	5	2.1
	Environmental Health	5	2.1
	Medical doctor	7	2.9
	Head nurse	16	6.6
	Supervisor	11	4.5
	Faculty member	1	0.4

**Fig. 1:**
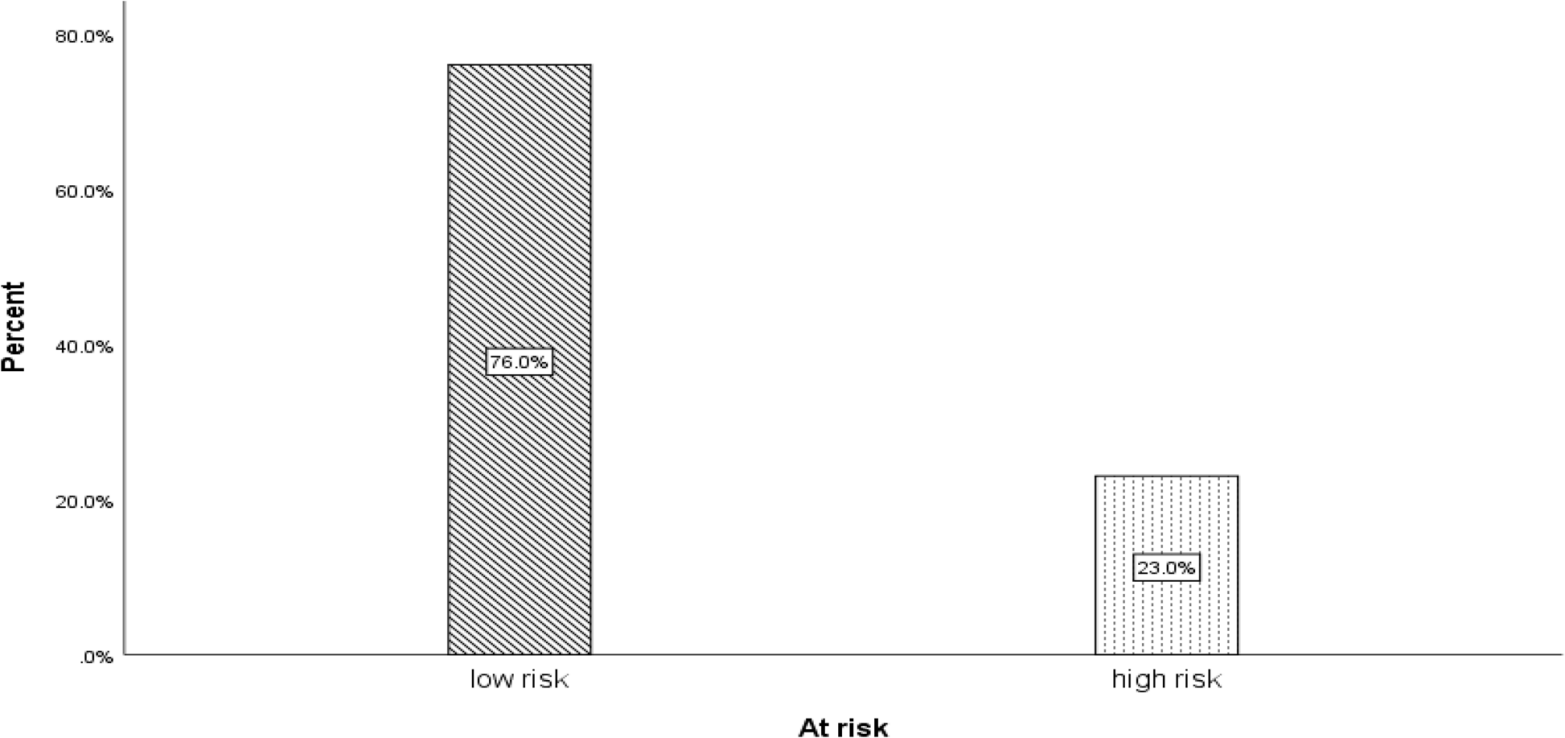
Prevalence of occupational exposure of healthcare workers to COVID-19

The results showed that all participants in the study had occupational exposure to the COVID-19 virus. According to the results, in 243 healthcare workers in Qazvin province, 186 people (76.5%) were at low risk, and 57 people (23.5%) were at high risk of COVID-19 disease, as shown in Fig. 1.

Table 2 examines the domains of occupational exposure to COVID-19 in healthcare workers in two groups of low-risk and high-risk of infection, using an independent t-test. The results showed a statistically significant difference between the mean scores of all domains in the two groups (*p*-value < 0.05).

**Table 2:** Mean and standard deviation of occupational exposure areas with COVID-19 in healthcare workers

Variable	Group		*p*-value
	**low risk**	**high risk**	
Health worker exposure type with a confirmed COVID-19 patient	3.55 ± 0.66	3.26 ± 0.76	0.006
Health worker interaction type with a confirmed COVID-19 patient	26.58 ± 28.55	47.82 ± 61.62	0.014
Health worker activities performed on a confirmed COVID-19 patient	14.68 ± 3.49	16.07 ± 3.11	0.008
Adherence to IPC during health care interactions	14.81 ± 3.93	17.10 ± 4.36	0.001
Adherence to IPC when performing aerosol generating procedures	15.54 ± 4.90	20.43 ± 4.98	0.001
Accidents with biological material	5.85 ± 0.79	5.36 ± 1.14	0.015

In the domains of type of healthcare workers’ exposure to a confirmed COVID-19 patient and accidents with biological material, the mean score of the low-risk group was higher than the high-risk group. That is, the participants who have these two domains have a lower risk of contracting Covid-19. However, in the domains of type of interaction with a confirmed COVID-19 patient, health worker activities performed on a confirmed COVID-19 patient, adherence to IPC during health care interactions, and adherence to IPC when performing aerosol-generating procedures, the mean score of the high-risk group is higher than the low-risk group. That is, the participants who have these four domains have high risk of contracting Covid-19.

Table 3 examines the relationship between demographic variables and the risk of COVID-19 disease infection in healthcare workers using the mean and frequency distribution (percentage). Using the Chi-square test (Fisher), no statistically significant difference was observed in the frequency (the percentage) of variables of gender, city of service, type of health professional, and level of education in the two groups of low-risk and high-risk of COVID-19 (*p*-value < 0.05). However, in examining the mean scores of age and work experience of workers in the two groups using an independent t-test, a statistically significant difference was observed (*p*-value < 0.05), in which younger people with low work experience were in the high-risk group.

**Table 3:** Relationship between demographic variables and COVID-19 risk in healthcare workers

Variable	Group	low risk (186)	high risk (57)	0R	*p*-value
		**Frequency (Percentage)**			
**Gender**	Male	65 (34.9)	18 (31.6)		*P* = 0.639*
	Female	121 (65.1)	39 (68.4)	1.164	
**City**	Center	109 (58.6)	46 (80.7)		*P* = 0.005**
	East	56 (30.1)	4 (7)	0.169	
	West	14 (7.5)	5 (8)	0.846	
	South	7 (3.8)	2 (3.5)	0.677	
**Highest level of qualification**	Bachelor	168 (90.3)	43 (75.4)		*P* = 0.004*
	Master	18 (9.7)	14 (24.6)	3.039	
**Type of exposure with COVID-19**	Direct	175 (94.1)	57 (100)	-	*P*= 0.049**
	Indirect	11(5.9)	0 (0)		
**Quantitative variables**		**Mean ± SD**		***p*-value**	
**Age**		35.56 ± 7.75	32.94 ± 6.76	*P* = 0.022***	
**Work experience**		11.72 ± 7.17	9.35 ± 6.88	*P* = 0.029***	

^*^ Chi-square test

^**^Fisher test

^***^Independent t test

## Discussion

This study aimed to investigate the healthcare workers’ exposure to COVID-19 in Qazvin province in 2020. Since healthcare workers provide direct care for COVID-19 patients, they are at the COVID-19 infection risk [20]. This study showed that some healthcare workers were at low risk and some at high risk of COVID-19 in Qazvin province. In line with the present study, Albaqawi et al. (2021) concluded that nurses working in Saudi Arabia’s corona sectors had a high exposure rate to COVID-19. However, the risk of COVID-19 infection was low in these nurses [9]. Also, Ashinyo et al. (2020) on Ghanaian healthcare workers and Ng et al. (2020) on Chinese healthcare workers reported that these individuals were at low risk of infection to COVID-19 [10, 21]. In line with the current study, Savini et al. (2017) concluded that workers caring for Ebola patients in Guinea were at low risk of Ebola virus infection [22]. Based on the findings of these studies, healthcare workers are highly exposed to COVID-19. Still, the risk of infection is reduced by using standard methods such as personal protective equipment, hand hygiene, etc. [21].

According to the results, in health worker exposure type with a confirmed COVID-19 patient and accidents with biological material, the mean score of the low-risk group is higher than the high-risk group. Nevertheless, in health worker interaction types with a confirmed COVID-19 patient, health worker activities performed on a confirmed COVID-19 patient, adherence to IPC during health care interactions, and adherence to IPC when performing aerosol-generating procedures, the average score of the high-risk group was higher than the low-risk group. In line with the present study, Albaqawi et al. (2021) on Saudi nurses, Ashinyo et al. (2020) on Ghanaian healthcare workers, and Mick and Murphy (2020) on Chinese healthcare workers noted that aerosol-producing procedures are high-risk medical activities that put healthcare workers at high risk of infection to the COVID-19 [9, 10, 23]. Therefore, the WHO recommends using personal protective equipment such as gloves, masks, face shields, goggles, gowns, and waterproof aprons during aerosol-producing procedures such as coughing, sneezing, and suctioning endotracheal secretions in a confirmed or suspected COVID-19 patient [10, 19].

In line with the present study, Albaqawi et al. (2021) reported that nurses working in Saudi Arabia were less likely to follow the principles of infection prevention and control during care activities for COVID-19 patients, such as using personal protective equipment and timely replacement, wash handing principles before and after providing care and touching patients and their surroundings. Hence, they are more at risk for COVID-19 infection [9].

Lack of knowledge among healthcare workers about the benefits of using personal protective equipment, severe sweating when using masks and goggles, and low vision when using face shields and glasses may prevent people from using personal protective equipment continuously. Therefore, according to WHO recommendations, continuous training of healthcare workers on infection control and prevention measures and appropriate personal protective equipment can protect exposed healthcare workers from infection with COVID-19 [9, 10].

The present study's findings indicate a statistically significant difference in the mean scores of age and work experience of workers in the two groups using an independent t-test (*p*-value < 0.05). Workers with low age and work experience are in the high-risk group, and workers with high age and work experience are in the low-risk group. Based on the present study results, workers with higher age and work experience have more experience dealing with infectious diseases. They have received continuous training on infection prevention and control principles over the years of service. They have obtained the necessary skills to adapt to stressful epidemic conditions. They also have fewer shifts than younger workers. On the other hand, due to their high work experience, they care less for critical and infectious patients or serve as supervisors in clinical departments, so they are at lower risk of infection to COVID-19 than younger workers with less work experience.

## Conclusion

Despite WHO strict guidelines, many healthcare workers are at risk of COVID-19 infection. According to the results in adherence to IPC during healthcare interactions and adherence to IPC when performing aerosol-generating procedures, the average score of the high-risk group is higher than the low-risk group. Younger workers with lower work experience were also at higher risk for COVID-19 infection. Therefore, health managers, planners, and policymakers can revise policies, provide appropriate and timely personal protective equipment, plan for ongoing training of workers on infection prevention and control principles, and closely monitor these principles can help solve these challenges.

### Rigor of study

Considering that the present study was performed on the Qazvin province health workers, generalizing the results to other populations should be made with caution. The use of self-report tools was another limitation of this study. Participants may not have a clear understanding of the implications of this study for answering related questions, so we recommend that more extensive qualitative studies and open interviews be conducted.

## Data Availability

The datasets used and/or analyzed during the current study available from the corresponding author on reasonable request. The entire dataset is in Farsi language. The Data can be available in English language for the readers and make available from the corresponding author on reasonable request.

## Abbreviations

WHO: World Health Organization
IPC: Infection prevention and control
SARS-COV-2: Severe acute respiratory syndrome coronavirus 2
